# Genetic structure of *Culex tritaeniorhynchus* (Diptera: Culicidae) based on COI DNA barcodes

**DOI:** 10.1080/23802359.2021.1911711

**Published:** 2021-04-15

**Authors:** Gui-Lin Xie, Xin-Ran Ma, Qi-Yong Liu, Feng-Xia Meng, Chao Li, Jun Wang, Yu-Hong Guo

**Affiliations:** a College of Life Science, Northeast Agricultural University, Harbin, China; b State Key Laboratory of Infectious Disease Prevention and Control, Collaborative Innovation Center for Diagnosis and Treatment of Infectious Diseases, National Institute for Communicable Disease Control and Prevention, Chinese Center for Disease Control and Prevention, Beijing, China

**Keywords:** *Culex tritaeniorhynchus*, COI, genetic structure, China

## Abstract

*Culex tritaeniorhynchus* Gile is a major vector of Japanese encephalitis in China. The population genetics study is crucial as it helps understanding the epidemiological aspects of mosquito-brone diseases and improving vector control measures. Here, the genetic population structure of *C. tritaeniorhynchus* in the mainland China were estimated using the cytochrome *c* oxidase subunit 1 (COI) DNA barcodes region. 485 individuals of *C. tritaeniorhynchus* were collected from 38 sampling sites in 21 geographic populations in the mainland China. In total, 485 sequences were used to explore the population structure and genetic diversity. The results showed that the populations of *C. tritaeniorhynchus* had high haplotype diversity (Hd = 0.98, with 303 haplotypes), low nucleotide diversity (*p* = 0.02245) and high gene flow (*Nm* = 47.11) with two maternal lineages and four groups. An AMOVA indicated that 98.8% of the total variation originated from variation within populations. In addition, the population genetic structure exhibited by *C. tritaeniorhynchus* filling the vacant of the genetic structure in the mainland China. Human activities may also assist mosquito movement and migration. Gene flow among the populations of *C. tritaeniorhynchus* can facilitate the spread of insecticide resistance genes over geographical areas, and it will be a challenging for controlling the populations.

## Introduction

*Culex tritaeniorhynchus* Gile is the principal vector of Japanese encephalitis (JE). JE is a mosquito-borne zoonosis caused by infection with Japanese encephalitis virus (JEV). JE usually manifests as mild central nervous symptoms, primarily in children and adolescents. In addition, it can also cause sow abortion and equine encephalitis, bringing serious economic losses (Zheng et al. 2012; Yun and Lee 2014; Mansfield et al. 2017). All provinces of China, except Xinjiang and Qinghai, have reported cases of JE (Ren et al. 2017). Although the JE vaccine immunization policy has been implemented in China, it is common to fail to complete the immunization program with JE vaccine for children in remote areas, especially in rural areas (Liu et al. 2018). Although immunization is considered to be the most important measure to prevent JE, there are still children infected with JE virus after vaccination (Zhang et al. 2011), and in recent years, there have been adult JE outbreaks in Shanxi in 2006 and Hebei in 2013 (Longbottom et al. 2017). *C. tritaeniorhynchus* also has the ability to transmit some other human and animal viral diseases (Hayes et al. 1980; Sallam et al. 2013). In view of the above, China still is facing a growing public health threat.

Vector capacity is controlled by genetic factors that affect the mosquito’s ability to transmit pathogen (Donnelly et al. 2002; Gorrochotegui-Escalante et al. 2002). Therefore, understanding the dynamics of *C. tritaeniorhynchus* populations, particularly genetic structure and genetic diversity, is important for the prevention and control of mosquito-borne diseases. The cytochrome *c* oxidase subunit 1 (COI) DNA barcodes region is considered as a valuable and reliable diagnostic tool for studying the genetic structure and genetic diversity of mosquitoes (Zawani et al. 2014; Zouré et al. 2020). The population genetics study is important for improving the vector control measures, primarily the genetic control, to prevent or reduce the epidemic impacts. This research aims to investigate the population genetics structure of *C. tritaeniorhynchus* using the COI DNA barcodes region from 21 geographic populations of China for providing significant information on the population dynamics of a species.

## Materials and methods

### Taxa sampling

**Figure 1.**
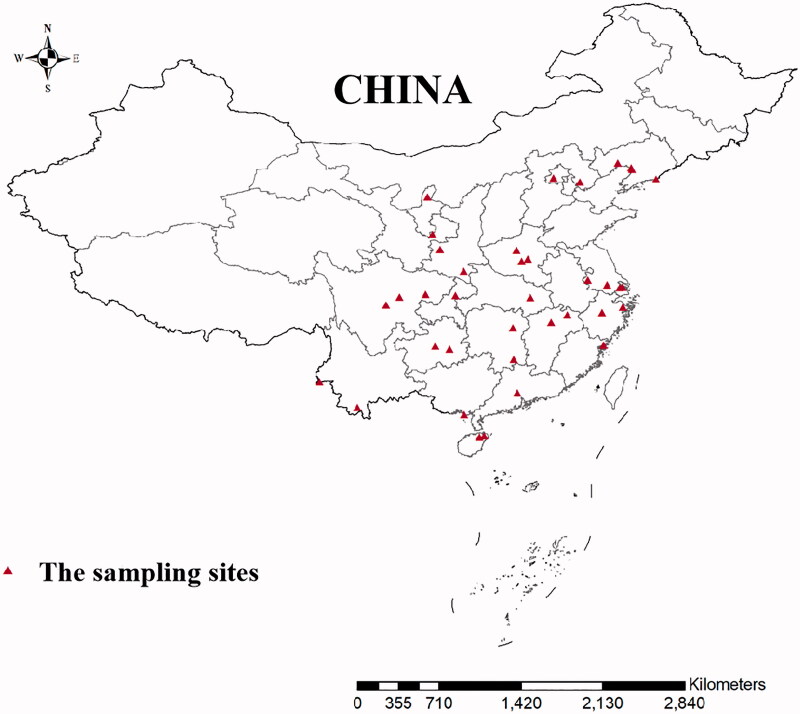
Sampling sites of 21 *Culex tritaeniorhynchus* geographic populations in the mainland of China.

Mosquitoes were collected from the following 38 sampling sites in 21 geographic populations: Liaoning (LN), Zhejiang (ZJ), Shanghai (SH), Jiangsu (JS), Fujian (FJ), Hebei (HB), Jiangxi (JX), Beijing (BJ), Hubei (HUB), Henan (HEN), Guangdong (GZ), Hunan (HUN), Hainan (HAN), Guangxi (GX), Shaanxi (SX), Chongqing (CQ), Guizhou (GZ), Gansu (GS), Ningxia (NX), Sichuan (SC), Yunnan (YN) in the mainland of China, July to September from 2014 to 2020 (Figure 1). Adult female mosquitoes were captured using light traps and preserved in 95% ethanol and stored at 4 °C. Adults in the traps were collected for morphological identification according to morphological criteria using the key developed (Lu 1997). We also uesd 9 specimens of the *C. tritaeniorhynchus* from Singapore (KF564730.1-KF564732.1) and India (KM350664.1-KM350669.1).

### DNA extraction and polymerase chain reaction amplification

Genomic DNA was extracted from head and thorax with a Micro Tissue Genomic DNA Extraction Kit (BioTeke, Wuxi, China) and a DNA/RNA Extractor-32 system (BioTeke). A 658-bp fragment of COI region was PCR-amplified using the Folmer primers LCO1490 and HCO2198 (Folmer et al. 1994). DNA amplification was conducted in 25-μl reactions using 12.5 μl of PCR mix (TsingKe Co. Ltd Beijing, China), 1.0 μl of 10 μM each primer, 1.5 μl of template DNA, and 9 μl of ddH_2_O. The amplification program consisted of one cycle at 94 °C for 5 min, followed by 35 cycles at 94 °C for 30 s, 55 °C for 30 s, and 72 °C for 1 min, and one cycle at 72 °C for 5 min (Beebe 2018). PCR products were electrophoresed on 1.0% agarose gel and then sent for sequencing at TsingKe Co., Ltd. (Beijing, China).

### Data analysis

Raw sequences were edited and assembled using SeqMan version 7.1.0 (Swindell and Plasterer 1997) and aligned using the Muscle algorithm in MEGA X (Sudhir et al. 2018). MEGA X was used to calculate the genetic distance between populations. MEGA X was utilized to select Tamura 3-parameter (T93) model and gamma distributed (G) rates among sites for construction of a maximum likelihood (ML) tree. A neighbor joining (NJ) tree based on Kimura 2-parameter model was also generated using MEGA X. We also used the sequences from GenBank of *Culex* species inculding *C. restuans* (GU908095.1), *C. interrogator* (JX259909.1), *C. tarsalis* (GU908102.1), C. *salinarius* (GU908096.1), *C. nigripalpus* (JX259910.1), and *C. pipiens pallens* (FN395206.1), C. *pipiens* (GU908084.1), *C. quinquefasciatus* (HQ398883.1), C. *quinquefasciatus* (GQ165798.1) were included as out-group taxa. A mtDNA COI haplotype network was constructed using the TCS inference method (Clement et al. 2000) implemented in POPART (Leigh and Bryant 2015). Analysis was performed using DnaSP V.5.10.1 to reveal the number of haplotypes, haplotype diversity, nucleotide diversity, and variable sites (Librado and Rozas 2009). The partitioning of genetic variation within and among populations was calculated via the analysis of molecular variance (AMOVA) with 1,000 permutations implemented in Arlequin v. 3.0 (Excoffier et al. 2017).

## Results

The COI gene amplified 658 bp sequence with 160 variable sites, 94 parsimony-informative sites and 66 were singleton variable sites for *C. tritaeniorhynchus*. The average percentages of T, C, A, and G nucleotides were 39.1%, 15.5%, 29.6%, and 15.8% respectively, the content of A + T was higher than that of G + C, which showed the use of nucleotides was favorable. The overall genetic diversity of *C. tritaeniorhynchus* are summarized in Table 1. High haplotype diversity (Hd = 0.98) and low nucleotide diversity (*p* = 0.02245) were showed in *C. tritaeniorhynchus* populations. A total of 303 haplotypes were identified which the most common haplotype was H2, H55 and H130. Genetic distance was calculated based on the Kimura 2-parameter model. The results showed that the intraspecific genetic distance was 0.000–0.069 among 21 *C. tritaeniorhynchus* population. The average genetic distances between intraspecific genetic distance was 0.024.

**Table 1.** Genetic diversity of 21 geographic populations based on COI barcode.

Samples sites	*n*	H	S	Hd	p	K
BJ	15	13	40	0.971	0.00974	6.40952
CQ	6	6	40	1.000	0.3040	20.00000
FJ	13	13	23	1.000	0.00758	4.98718
GD	5	4	9	0.900	0.00547	3.60000
GS	12	11	46	0.985	0.02888	19.00000
GX	24	19	52	0.960	0.01228	8.07971
GZ	29	26	57	0.990	0.02307	15.18227
HEB	15	13	46	0.981	0.02841	18.6954
HUB	7	5	8	0.905	0.00463	3.04762
HAN	24	20	33	0.964	0.00651	4.28261
HUN	38	29	63	0.979	0.02603	17.13087
JS	27	22	52	0.983	0.02736	18.00285
JX	15	11	51	0.905	0.02908	19.13333
HEN	48	36	59	0.973	0.01973	12.98138
NX	13	11	40	0.962	0.02326	15.30769
SC	39	32	53	0.970	0.02358	15.51552
SH	29	19	64	0.904	0.03007	19.78818
SX	20	18	50	0.989	0.02442	16.06842
LN	57	46	67	0.984	0.02016	13.26504
YN	23	23	53	1.000	0.01379	9.07115
ZJ	26	23	58	0.982	0.02253	14.82462

*n*: Number of samples; H: Number of haplotypes; S: Number of variable sites; Hd: Haplotypes diversity; p: Nucleotide diversity; K: Average number of nucleotide differences.

**Figure 2.**
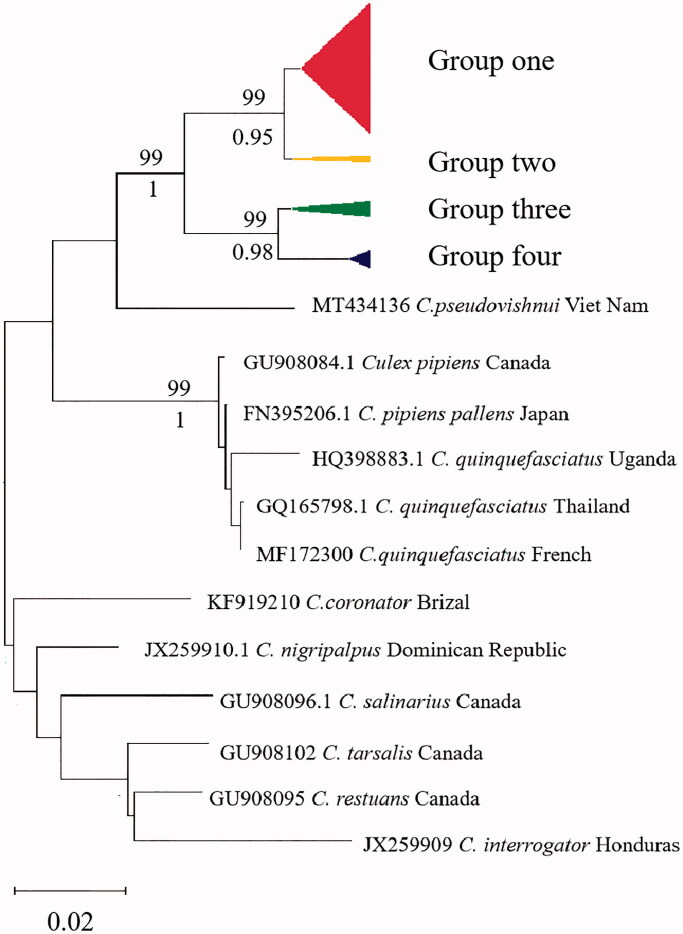
Neighbor-joining tree based on COI barcodes region of *C. tritaeniorhynchus*. Bootstrap support (1,000 replicates) of nodes from NJ tree and ML tree are indicated above and below the branches, respectively. Only nodes with BS > 70% are labeled.

**Figure 3.**
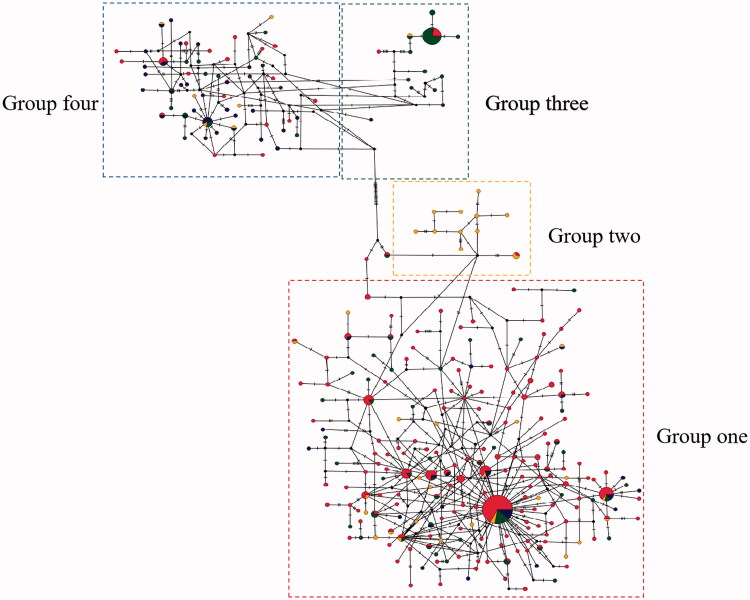
Haplotype network based on COI barcodes of *Culex tritaeniorhynchus*. Node sizes are proportional to haplotype frequencies. Lines linking the nodes are proportional to the mutation steps.

The AMOVA results are displayed in Table 2. The inheritance within populations accounted for 98.8% of the total genetic variation, suggesting that most of the population variation in *C. tritaeniorhynchus* was due to genetic variation within populations. According to the topology of NJ tree and ML tree, the phylogenetic tree showed the clustering of these samples into two maternal lineages and four groups, group one includes Singapore, LN, FJ, HB, BJ, HEN, GZ, HUN, HAN, GX, HUB, CQ, SC, group two includes India, YN, GZ, group three includes JX, JS, ZJ, SH, group four includes GS, NX, SX (Figure 2). The results were further supported by haplotype network based on COI gene (Figure 3). The 21 *C. tritaeniorhynchus* populations distributed in the mainland China showed a certain degree of genetic differentiation and a great deal of gene flow (*Fst* = 0.01203, *Nm* = 47.11).

**Table 2.** AMOVA analysis of *C. tritaeniorhynchus* populations based on COI gene fragments.

Source of variation	d.f.	Sum of squares	Variance components	Percentage of variation
Among groups	3	2.461	0.00276Va	0.56
Among populations within groups	17	9.455	0.00315Vb	0.64
Within populations	464	225.229	0.48541Vc	98.80
Total	484	237.144	0.49132	
Fixation Indices	*Fst* = 0.01203, *Nm* = 47.11			

## Discussion

This is the first extensive study of *C. tritaeniorhynchus* in the mainland China with sampling spanning many of provinces. It was found that *C. tritaeniorhynchus* with high haplotype diversity and low nucleotide diversity, we assumed that *C. tritaeniorhynchus* population had experienced a bottleneck effect, accompanied by rapid population expansion and accumulation of variation (John 2010). The degree of differentiation among different populations of *C. tritaeniorhynchus* was small and gene flow was frequent (*Fst* = 0.01203, *Nm* = 47.11). AMOVA analysis showed that the genetic differentiation of *C. tritaeniorhynchus* was mainly between individuals within the population (98.80%), and there was little difference between different populations (1.20%). This may due to the long flight distance of *C. tritaeniorhynchus*, with an average maximum flight distance of 2.2 km and a maximum flight distance of 7.5 km (Verdonschot and Besse-Lototskaya 2014), which may increase gene exchange among different populations. The population of *C. tritaeniorhynchus* in Hainan Island, China also showed high haplotype diversity (Li et al. 2020), which was basically consistent with the results of this study. The *C. tritaeniorhynchus* in this study came from different geographical populations. The average genetic distances between intraspecific genetic distance was 0.024.In diptera family, 4–7% of the genetic distance is normal intraspecie variation and morphologically within the same species, while the genetic distance of cryptic species or pseudocryptic species are usually higher than 13% (Lin et al. 2015).

According to the topology of NJ tree and ML tree, COI sequences of *C. tritaeniorhynchus* revealed that there were two maternal lineages and four groups in the mainland China. This study revealed that most geographically closer populations were genetically similar and were included in the same sub cluster except group one. The sites in group one are located in Singapore and the north, central and south of China. Human activities may also assist mosquito movement and migration by transporting scarp tires, also by moving water-holding containers (Md Naim et al. 2020). It indicates that *C. tritaeniorhynchus* from different regions had more gene flow and clustered into a single group. The geographic groups of Gansu (GS), Ningxia (NX) and Shannxi (SX) are grouped into one branch, NX was in the north of China which occurred an outbreak of JE in 2018 (Liu et al. 2020). Thus, the gene flow of *C. tritaeniorhynchus* may increase the risk of JE in these areas. With the movement of people and trade, it will enhance the gene flow between adjacent areas which leads to the formation of groups three and four. *C. tritaeniorhynchus* is the main vector of JE with a wide distribution in China. Except the use of bed nets, window screens and repellent, insecticides are also effective for vector control. Gene flow among the populations may assist the spread of insecticide resistance genes over geographical areas (Barnes et al. 2017), it will be a challenging for controlling the populations. Moreover, our study fills the genetic structure vacant of *C. tritaeniorhynchus* population in the mainland China which providing guidance for vector control of JE disease.

## Conclusion

In this study, *C. tritaeniorhynchus* has a certain pedigree and geographical structure in the mainland China. The results showed that the populations of *C. tritaeniorhynchus* had high haplotype diversity, low nucleotide diversity and high gene flow with two maternal lineages four groups. Human activities and the use of pesticides will affect the mosquito populations which may develop insecticide resistance gene and a challenging for controlling the mosquito vector populations.

## Data Availability

The data that support the findings of this study are available in the National Center for Biotechnology Information (NCBI) at [https://www.ncbi.nlm.nih.gov/], reference number [MW488441-MW488925]
